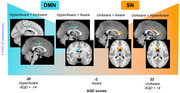# Functional connectivity dysregulation across the awareness continuum in Alzheimer's Disease: from hypernosognosia to anosognosia

**DOI:** 10.1002/alz70857_101107

**Published:** 2025-12-25

**Authors:** Manuela Tondelli, Daniela Ballotta, Riccardo Maramotti, Chiara Carbone, Chiara Gallingani, Giuseppe Pagnoni, Annalisa Chiari, Giovanna Zamboni

**Affiliations:** ^1^ Università di Modena e Reggio Emilia, Modena, Italy; ^2^ University of Modena and Reggio Emilia, Modena, Italy; ^3^ University of Ferrara, Ferrara, Italy; ^4^ Neurologia, Azienda Ospedaliero Universitaria di Modena, Modena, Italy; ^5^ Azienda Ospedaliero Universitaria di Modena, Modena, Italy

## Abstract

**Background:**

Recent evidence suggests that anosognosia in the Alzheimer's disease (AD) is associated with a dysregulation of the of three large‐scale networks, namely the Default‐Mode (DMN), the Salience (SN), and the Fronto‐Parietal (FPN) Network. Here, we further investigate if this functional connectivity dysregulation shows a different trajectory across the continuum between hypernosognosia (i.e. increased awareness of cognitive function) and anosognosia (i.e. reduced awareness of cognitive function).

**Method:**

We evaluated 60 patients with mild cognitive impairment (MCI) and Alzheimer's disease (AD) dementia using functional MRI (fMRI) and neuropsychological assessments, including the Anosognosia Questionnaire for Dementia (AQ‐D). Discrepancy scores from the AQ‐D, reflecting differences between patient and caregiver evaluations, were used to categorize participants into three groups: hyperaware (hyAW; AQD < ‐14), unaware (uAW; AQD > 14), and aware (AW; ‐14 ≤ AQD ≤ 14). Resting‐state fMRI data were analyzed using Independent Component Analysis (ICA) to investigate functional connectivity within the DMN, SN, and FPN, with group‐wise comparisons conducted subsequently.

**Result:**

Hyperaware (hyAW) subjects exhibited increased DMN functional connectivity in the posterior cingulate cortex compared to both uAW and AW groups, and in the midcingulate cortex compared to AW subjects. Unaware (uAW) subjects displayed heightened SN functional connectivity in the anterior cingulate cortex relative to hyAW participants, and in the anterior cingulate, anterior insula, and basal ganglia relative to AW group. Additionally, uAW subjects showed enhanced FPN functional connectivity in the inferior frontal gyrus compared to hyAW subjects.

**Conclusion:**

These findings confirm that awareness levels within the AD continuum are associated with imbalances in the functional connectivity of the DMN, SN, and FPN. the recruitment of these networks follows a gradient across the awareness spectrum, providing critical insights into the neural underpinnings of awareness disruptions in Alzheimer's disease.